# Idiopathic Acquired Hemophilia A with Undetectable Factor VIII Inhibitor

**DOI:** 10.1155/2014/484563

**Published:** 2014-05-14

**Authors:** Nicholas B. Abt, Michael B. Streiff, Christian B. Gocke, Thomas S. Kickler, Sophie M. Lanzkron

**Affiliations:** ^1^Department of Medicine, Johns Hopkins University School of Medicine, Baltimore, MD, USA; ^2^Division of Hematology, Department of Medicine, The Johns Hopkins Hospital, 1830 E. Monument Street, Suite 7300, Baltimore, MD 21205, USA; ^3^Department of Pathology, The Johns Hopkins Hospital, 1800 Orleans Street, Sheikh Zayed Tower B1-1065, Baltimore, MD 21287, USA

## Abstract

*Objective*. We present the case of a 73-year-old female, with no family or personal history of a bleeding disorder, who had a classic presentation for acquired hemophilia A. Factor VIII activity was low but detectable and a factor VIII inhibitor was undetectable. *Methods*. The patient's plasma was comprehensively studied to determine the cause of the acquired coagulopathy. Using the Nijmegen modification of the Bethesda assay, no factor VIII autoantibody was measureable despite varying the incubation time from 1 to 3 hours. *Results*. The aPTT was prolonged at 46.8 seconds, which did not correct in the 4 : 1 mix but did with 1 : 1 mix. Using a one stage factor VIII activity assay, the FVIII activity was 16% and chromogenic FVIII activity was also 16%. The patient was treated with recombinant FVII and transfusion, significantly reducing bleeding. Long-term therapy was initiated with cyclophosphamide and prednisone with normalization of FVIII activity. *Conclusions*. Physicians can be presented with the challenging clinical picture of an acquired factor VIII inhibitor without a detectable inhibitor by the Bethesda assay. Standard therapy for an acquired hemophilia A should be considered.

## 1. Introduction

Acquired hemophilia A (AHA) is caused by autoantibodies, usually polyclonal IgG1 and IgG4 subtypes, acting as inhibitors against factor VIII [1]. Acquired factor VIII deficiency usually presents as spontaneous, unanticipated hemorrhage. If the bleeding is not controlled in a timely manner, the deficiency can be life threatening. Hemophilia A has an incidence of 0.2 to 1.48 cases per million people per year [2]. Typical age of presentation is a biphasic distribution of 20 to 30 years and greater than 60 years. The acquired inhibitor has been linked to a number of causes including pregnancy, drugs, malignancies, autoimmune disorders, collagen vascular disorders, respiratory disorders, and infections. Even with these linkages, over 50% of cases are idiopathic in etiology [3].

A patient presenting with unusual bleeding and no family history of bleeding, along with an inexplicable prolonged activated partial thromboplastin time (aPTT) suggests an acquired hemophilia A. These hemorrhages can be manifest in the skin, soft tissue, muscle, and mucous membranes. Importantly, before a diagnosis of factor VIII inhibitor can be made, other causes of a prolonged aPTT should be ruled out, including antiphospholipid antibodies and factor XII deficiency. Additionally, heparin therapy, factor deficiencies, or inhibitors to other components of the intrinsic pathway are still on the differential diagnosis. Mixing studies are performed to elucidate if an inhibitor, whether specific or nonspecific, is present [3].

We present a patient with a classic clinical presentation of an acquired inhibitor with a difficult diagnostic dilemma.

## 2. Case Report

A 73-year-old female, without a prior history of bleeding or hemophilia, presented with difficult to control bleeding, large ecchymoses over her body, petechiae, and hemarthrosis over the prior 2 months. She was in her typical state of health until she fell in her home and landed on her left hip in early November, 2012. A large ecchymosis extended both distally and proximally from the site of injury. Over the next month, she noticed spontaneous bleeding, after minor or no trauma, over her body to include bilateral arms, right thigh, and left ankle. Both her right knee and left ankle became swollen and were limited in their range of motion. Several days prior to admission to an outside hospital, the patient reported increasing dyspnea on exertion, with new right thigh and tongue swelling. She was subsequently discharged, readmitted, and transferred to Johns Hopkins Hospital.

The past medical history revealed hypothyroidism, vitiligo, Raynaud phenomena, hypertension, coronary artery disease, and a rectal polyp. Previous surgeries included an appendectomy in 1958, herniated disc surgery in 1983, bare-metal stenting in 2007, and a polyp removal in 2012. The 6 cm rectal polyp was removed in August, 2012, which was complicated by postoperative bleeding after being discharged the same day, but the patient did not require blood transfusion or hospitalization at that time. All previous surgeries were uncomplicated without incident of excess blood loss. The family history was absent of bleeding diatheses. She previously drank significant amounts of alcohol but had cut down in 2012 and had a smoking history of 50 pack-years.

The outside hospital initially evaluated her in December and at that time she had a normal PT, fibrinogen, and platelet count. The aPTT level was elevated to 50–60 seconds. Erythrocyte sedimentation rate was reported as 54 mm/hr and C-reactive protein of 1.1 mg/dL. Coombs' test was negative with a normal serum protein electrophoresis and haptoglobin level.

When she arrived to JHH, she was found to have a low factor VIII level at 16% with active bleeding. Despite this finding we did not measure any inhibitory activity in her plasma using Nijmegen modification of the Bethesda's assay. It is of note that we found in the aPTT evidence of an inhibitor. Immediately, the 4 : 1 mixed aPTT was delayed at 34.3 seconds (reference range: 23.3–30.3 s). After 2 hours, the 4 : 1 mixed aPTT continued to be prolonged at 39.7 seconds. All of her other factor activity levels were normal: FII: 138%, FV: 192%, FVII (measured after recombinant FVIIa given): 1212%, FIX: 137%, FX: 190%, FXI: 90%, and FXII: 60%. Because acquired inhibitors may have variable kinetics in inhibiting coagulation, we varied the incubation times in the Bethesda assay from 30 minutes to as long as 180 minutes and detected no inhibition. Additionally, the liver function tests were within normal limits.

Chromogenic assay revealed a significantly low factor VIII antigen of 16%. Dilute Russell viper venom time was normal at 37 seconds. A heparinase test did not shorten the aPTT. Euglobulin lysis (>60 mins), von Willebrand antigen (>150%), and ristocetin cofactor (>149%) were all normal. Both an antinuclear antibody screen and hepatitis B and C studies were negative. Serum immunoglobulin assay showed a slightly low IgG level of 629 mg/dL with normal IgA and IgM levels of 147 mg/dL and 161 mg/dL, respectively. The serum protein electrophoresis showed no monoclonal gammopathy. The remainder of the work-up laboratory data can be found in Table 1.

**Figure 1:**
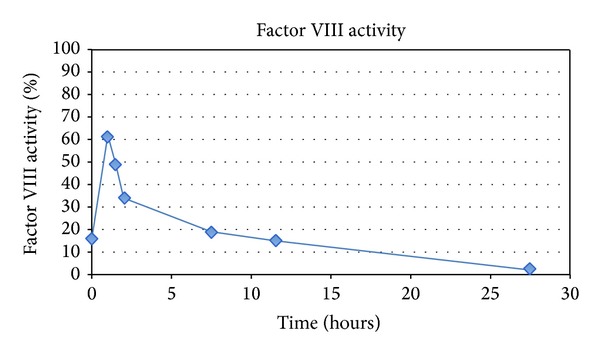
Serial factor VIII activity levels after recombinant factor VIII trial of 50 units/kg of factor VIII given at time 0.

Despite the negative Bethesda assay, her clinical presentation was consistent with the presence of an inhibitor. Thus, a trial of recombinant FVIII was given and serial FVIII activities were done to determine recovery time. Fifty units/kg of factor VIII was given and at 60 minutes FVIII activity was 62%. Subsequent drop-offs of the factor VIII activity are shown in Figure 1.

To control bleeding, recombinant activated factor VII (NovoSeven RT) was given every two hours for 24 hours. The patient's hemoglobin stabilized and she was started on prednisone at one mg/kg a day. Cyclophosphamide was initiated the same day at 100 mg/day. Over a period of 8 months, the factor VIII activity steadily increased to a maximum of 220% without any further bleeding complications. However, cyclophosphamide was stopped 38 days after initiation due to toxicity. Prednisone has been slowly tapered over the 8-month treatment course. The laboratory data, clinical course, and treatment can be followed in Table 2.

## 3. Discussion

Acquired hemophilia A is a rare hematologic disorder caused by factor VIII autoantibodies. The commonest presentation is unprovoked, spontaneous hemorrhage, which can be life threatening [2]. The mortality rate ranges from 8 to 22%, but rates are improving with enhanced hemostatic agents such as activated prothrombin complex concentrations and recombinant factor VIIa [4].

Diagnosing AHA is complicated because often times the patient does not have a personal or family history of a bleeding disorder. Additionally, the clinical depiction of AHA is different than a hereditary hemophilia. Patients with a factor VIII inhibitor tend to bleed into the skin, muscles, soft tissues, or mucous membranes while those with classic hereditary hemophilia hemorrhage into the joints [5].

The clinical suspicion of acquired hemophilia should prompt the clinician to order PT and aPTT studies. In acquired hemophilia, the PT is normal and the aPTT is prolonged. Often the aPTT corrects in a 1 : 1 mix without incubation but then becomes prolonged after 37-degree incubation. This is a phenomenon not seen in our patient. A one stage factor assay will show a low factor VIII activity, typically lower than 5 percent (0.05 IU/dL). The other factor activities will be normal. The diagnosis is confirmed using a formal Bethesda assay, ideally modified to detect low titer inhibitors. Autoantibody factor inhibitors of VIII are polyclonal in most cases, and have complex reaction kinetics. Consequently patients with acquired hemophilia often have residual circulating factor VIII activity. In our patient, despite modifying the incubation time to 30 minutes and extending to 3 hours, we were unable to detect any in vitro inhibition. The reason for this is unknown.

Typically, once autoantibody presence is confirmed, prompt immunosuppressive therapy should be initiated for autoantibody eradication. Typical treatment consists of 1/mg/kg/d of prednisone for 2 weeks, followed by a 6-week taper period, either alone or in combination with 1-2 mg/kg/d cyclophosphamide for up to 12 weeks. If prednisone or cyclophosphamide is contraindicated or ineffective in treatment, rituximab can be used [3]. This patient was treated as though an inhibitor was found.

Although no factor VIII inhibitor was detected using the Bethesda assay, our patient displayed characteristic signs of an inhibitor [6]. Importantly, despite the negative Bethesda assay, the mixing study indicated to the clinicians the possibility of an inhibitor. We were able to exclude heparin or an antiphospholipid antibody based upon specific testing. Most noticeably, exposure to factor VIII at 50 units/kg should have resulted in a 100% recovery of activity but the patient's levels were markedly lower. Moreover, the patient responded well to the standard treatment of a known acquired factor VIII antibody: FVIIa, cyclophosphamide, and prednisone. Theories as to why the AHA presented as an acquired factor VIII inhibitor without detection of an inhibitor are numerous. The antibody could be against the factor VIII receptor, not routinely tested in the Bethesda assay. Another possibility could be antibody against von Willebrand factor (vWF), which binds to and protects factor VIII from degradation [7]. However, her vWF levels were normal. Lastly, one wonders if she could be a carrier of a factor VIII deletion, the mutation associated with the highest risk of inhibitor formation [8]. This case is unique as the current literature does not describe nor explain the phenomenon of an undetectable factor VIII inhibitor in acquired hemophilia A.

## Figures and Tables

**Table 1:** Laboratory work-ups at the initial presentation and after 4 months of follow-up.

	Reference range	Initial presentation	4-month follow-up
aPTT (s)	23.3–30.3	44.2	NA
aPTT ratio (PAT/normal)	—	1.7	NA
Prothrombin time (s)	9.4–11.6	10	NA
D-dimer (mg/L fibrinogen equivalent units)	0.17–0.88	3.17	NA
Erythrocyte sedimentation rate (mm/hr)	1–30	45	NA
FVIII activity (%)	50–200	16	202
FVIII Inhibitor (Bethesda units/mL)	0-1	0.0	NA
Von Willebrand factor antigen (% of normal)	50–150	>150	NA
4 : 1 mix aPTT immediate (s)	23.3–30.3	34.3	NA
4 : 1 mix aPTT 2 hours (s)	26.1–36.0	39.7	NA
Euglobulin lysis (min)	>60	>60	NA
Ristocetin cofactor (%)	50–150	>149	NA
Serum IgG (mg/dL)	751–1560	629	NA
Serum IgA (mg/dL)	82–453	147	NA
Serum IgM (mg/dL)	46–304	161	NA
Antinuclear antibody screen	—	Negative	NA

**Table 2:** The laboratory data and clinical course of acquired hemophilia A.

	Day 1	Day 7	Day 31	Month 3	Month 6	Month 8
aPTT (s)	44.2	NA	NA	NA	NA	NA
aPTT ratio (PAT/normal)	1.7	NA	NA	NA	NA	NA
FVIII activity (%)	16	6	44	136	220	166
FVIII inhibitor (BU/mL)	0.0	NA	0.0	NA	NA	NA
Hemoglobin (g/dL)	8.5	10.0	12.2	14.6	15.1	14.3
Reticulocyte count (%)	6.4	NA	6.2	3.8	2.7	2.3
Absolute reticulocyte Count (×10^3^/*μ*L)	191.0	NA	209.0	154.0	117.0	98.2
Platelet (×10^3^/*μ*L)	267	322	180	201	150	209
Immunosuppressive	Prednisone: 80 mg/day PO cyclophosphamide: 100 mg/day PO		Prednisone: 60 mg/day PO cyclophosphamide: 100 mg/day PO*	Prednisone: 30 mg/day PO	Prednisone: 10 mg/day PO	None

*Cyclophosphamide stopped at day 38.
